# Clinical analysis of HPV58-positive cervical cancer

**DOI:** 10.1186/s13027-020-00303-w

**Published:** 2020-06-05

**Authors:** Mengjie Chen, He Wang, Yuejuan Liang, Li Li

**Affiliations:** Guangxi Medical University affiliated Cancer Hospital, NO.71 Hedi Road Qingxiu Square, Nanning City, Guangxi Province China

**Keywords:** Human papilloma virus, Cervical cancer, Uterine corpus invasion, Prognosis

## Abstract

**Objective:**

To study the clinical features of HPV58-positive cervical cancer.

**Methods:**

A retrospective analysis of 347 patients with HPV58- or HPV16 positive cervical cancer from the Department of Gynecology Tumor of Guangxi Medical University Affiliated Cancer Hospital was performed. Molecular hybridization was used to detect HPV genotypes. The clinical features, including age, pathology, and invasion, were compared between the HPV58 positive and HPV16 positive cervical cancer groups.

**Results:**

A total of 347 patients were eligible for this study, and the proportion of patients who were with poorly differentiated cancer (*P* = 0.015) was significantly higher in the HPV58 positive group. HPV58 positivity was an independent risk factor for poorly differentiated cancer [HR 2.156, 95% confidence interval: 1.167–3.984, *P* = 0.014]. The percentage of uterus corps invasion is significantly lower in HPV58 (*p* = 0.041), but HPV58 positivity is the independent risk factor for uterus corps invasion [HR = 3.985, 95% confidence interval: 1.066–14.893, *P* = 0.040]. The overall survival of HPV58-positive cervical cancer patients with uterine corpus invasion was significantly lower (*P* = 0.000). The age of patients in the HPV58-positive cervical cancer at advanced stage was significantly older (*P* = 0.045).

**Conclusions:**

HPV58-positive cervical cancer patients are at higher risk of poorly differentiated cancer and uterus corps invasion. The patients with HPV58 positive cervical cancer with uterus corps invasion may result a worse prognosis.

In the year 2015, there were 98,900 new cases of cervical cancer and 30,500 died [1]. High-risk human papilloma virus (HPV) subtypes play an important role in cervical cancer occurrence [2]. The most common high-risk HPV subtypes include the HPV16, 18, 58, 33, 45, 31, 52, 35, 59, 39, 51 and 56 [3]. In southwest China, the most common HPV genotype is HPV16 (54.67% of cases), followed by HPV58 (13.33% of cases) and HPV33 (5.33%) [4]. In the Zhejiang Province of China, HPV16 (62.1% of cases), HPV58 (10.52% of cases), HPV18 (10.95% of cases) and HPV52 (7.96%) are predominant [5]. Similarly, in the Yangtze River basin, HPV16 is the most common subtype (65.77% of cases), and the second most common is HPV58 (9.01% of cases) [6]. HPV genotypes are regionally distributed. In general, HPV16, HPV18, and HPV58 are dominant in China [3, 7]. In addition, the rate of infection with HPV58 is significantly higher in Asia than in America, Africa and Australia [8]. More than 80% of cervical cancer cases have one or more of the HPV genotypes, including the α-PV9 viruses (HPV16, 31, 33, 35, 52, 58 and 67) and the α-PV7 viruses (HPV18, 39, 45, 59,68 and 70) [9].

Because of the specificity of HPV58, the high infection rate in Asia and the lack of intensive research, this study discusses the clinical features of HPV58-positive cervical cancer to provide references for diagnosis and prognostication.

## Method

### Clinical data

A retrospective analysis of 347 patients with cervical cancer admitted in Department of Gynecology Oncology of Guangxi Medical University affiliated Cancer Hospital during 2010 to 2017 was performed. The patients were restaged according to the 2018 International Federation of Obstetrics and Gynecology (FIGO) staging system. Cases with metastatic cervical tumors, cervical cancer recurrence or with other existing malignant tumors were excluded. The research group was HPV58-positive cervical cancer, and the control group was HPV16 positive cervical cancer. Follow-up was performed by telephone. The patients with complete clinical data and follow-up data were eligible for the study. Patients who were lost to follow-up were excluded. The method used to categorize HPV genotypes was molecular hybridization.

### Administered therapy

All eligible patients had finished treatment. The treatment plans were made according to National Comprehensive Cancer Network (NCCN) guidelines (Table 1).

**Table 1:** Administration

Stage	Administered therapy	Adjunctive therapy
IA	Cone biopsy±extrafascial hysterectomy, or radical hysterectomy +pelvic lymph node dissection±para-aortic lymph node dissection	If postoperative pathology showed the presence of risk factors, adjunctive therapy was applied. Risk factors included: positive lymph nodes, positive surgical margins, parametrial invasion, tumor diameters over 4 cm, lymphovascular space invasion and deep interstitial invasion
IB	Radical hysterectomy +pelvic lymph node dissection±para-aortic lymph node dissection	
IIA	Radical hysterectomy +pelvic lymph node dissection±para-aortic lymph node dissection	
IIB-IVB	Pelvic external beam radiotherapy±concurrent chemotherapy	

### The evaluation of tumor invasion and metastasis

For patients who received surgery, tumor invasion and metastasis were evaluated by postoperative pathology reports and preoperative CT or MRI reports. For patients who did not undergo surgery, tumor invasion and metastasis were evaluated by pretreatment CT or MRI.

### Statistical analysis

Statistical analyses were performed using SPSS statistical software 22.0. Statistical descriptions were expressed as the mean ± standard deviation. Chi-square tests or Fisher’s test was used to calculate the differences between the data, and Student’s t-test or group rank sum tests were used to grade the data. The Kaplan-Meier method was used to construct a survival curve, and the statistical significance between the curves was assessed with the log-rank test for univariate analysis. Binary logistic regression models were used for multivariate analysis. All statistical analyses were two-tailed, and a P-value less than 0.05 was considered statistically significant. The survival curve pictures were made by PRISM software 8.0.

## Result

### The clinical features of 347 patients with cervical cancer

#### Clinical features

A total of 347 patients with cervical cancer were included in the study, Of these, 79 patients had HPV58-positive cervical cancer, a total of 74 cases were diagnosed with squamous cells carcinoma, 3 cases of adenocarcinoma and 2 cases of adenosquamous carcinoma. While 268 patients had HPV16 positive cervical cancer, including 256 cases of squamous cells carcinoma, 8 cases of adenocarcinoma and 4 cases of adenosquamous carcinoma (Table 2). The minimum age was 24 years old, the maximum age was 78 years old, and the median age was 52 years old. There were 63 cases and 176 cases of poorly differentiated cancer in the HPV58-positive and HPV16 positive cervical cancer groups, respectively, accounting for 79.75% and 65.67% of cases. The proportion of poorly differentiated carcinoma was significantly higher in HPV58-positive cervical cancer than in the HPV16 positive group (Table 3).

**Table 2:** HPV types and stages by hispathologytypes

	SCC		ADC/ASC		Total	
	N	%	N	%	N	%
HPV58	74	93.67	5	6.34	79	22.77
HPV16	256	95.52	12	4.48	268	77.23
Stage						
IA1	12	92.31	1	7.69	13	3.75
IA2	2	100	0	0	2	0.58
IB1	28	90.32	3	9.68	31	8.93
IB2	28	96.55	1	3.45	29	8.36
IB3	36	100	0	0	36	10.37
IIA1	20	100	0	0	20	5.76
IIA2	35	94.59	2	5.41	37	10.66
IIB	50	90.91	5	9.09	55	15.85
IIIA	4	100	0	0	4	1.15
IIIB	48	96.00	2	4	50	14.41
IIIC1r	38	97.44	1	2.56	39	11.24
IIIC2r	10	100	0	0	10	2.88
IVA	12	85.71	2	14.29	14	4.03
IVB	7	100	0	0	7	2.02

**Table 3:** The clinical feature of 347 patients with cervical cancer

	HPV58	HPV16	Z	P
Age			3.205	0.073
≤50 years old	27	122		
>50 years old	52	146		
Pathology type			0.449	0.503
SCC	74	256		
Non-SCC	5	12		
Differentiation			5.639	**0.018**
Poor	63	176		
Moderately/well	16	92		
Maximum diameter			0.311	0.577
<4 cm	28	86		
≥4 cm	51	182		
Lymph node metastasis			0.059	0.808
Yes	28	99		
No	51	169		
Bladder invasion			0.169	0.681
Yes	7	28		
No	72	240		
Rectal invasion			1.324	0.250
Yes	3	20		
No	76	248		
Vaginal invasion			0.103	0.748
Yes	25	90		
No	54	178		
Uterine corpus invasion			0.351	0.553
Yes	57	84		
No	22	184		
Distant metastasis			0.014	0.906
Yes	5	16		
No	74	252		

#### Risk factors for poorly differentiated cancer occurrence

Univariate analysis was performed to explore the risk factors associated with poorly differentiated cancer occurrence. The result showed that HPV58 positivity was an independent risk factor for poorly differentiated cancer occurrence [HR 2.156, 95% confidence interval: 1.167-3.984, *P*=0.014]. Additionally, squamous cells carcinoma was an independent risk factor for poorly differentiated cancer too, its risk for poorly differentiated cancer was 3.667 times than non-squamous cells carcinoma [HR 3.667, 95% confidence interval: 1.335-10.130, *P*=0.012]. Other factors were not included in the multiple logistics analysis (Table 4).

**Table 4:** Univariate and multivariate analyses of the risk factors for poorly differentiated cancer in 347 cases of cervical cancer

	Univariate analysis			Multivariate analysis		
	HR	95% CI	P	HR	95% CI	P
HPV58 positivity	3.652	1.060-12.584	**0.040**	2.156	1.167-3.984	**0.014**
Age<50 years old	1.140	0.719-1.807	0.578	/	/	/
SCC	3.382	1.251-9.141	**0.016**	3.677	1.335-10.130	**0.012**

Further analysis about the pathology type of squamous cells carcinoma was performed, the squamous cells carcinoma cases with HPV58 positivity was associated with increased risk for poorly differentiated cancer [HR7.038, 95% confidence interval: 1.067-46.437, *P*=0.043]. However, the HPV16 positive squamous cells carcinoma cases did not show increased risk for poorly differentiated cancer [HR 0.335, 95% confidence interval: 0.109-1.152, *P*=0.085] (Table 5).

**Table 5:** Univariate analysis of relationship of HPV genotypes and poorly differentiated cancer in cases with SCC

	HR	95% CI	P
HPV58 positivity	7.038	1.067-46.437	**0.043**
HPV16 positivity	0.335	0.109-1.152	0.085

#### Survival analysis of HPV58- and HPV16 positive poorly differentiated cancers

**Fig. 1:**
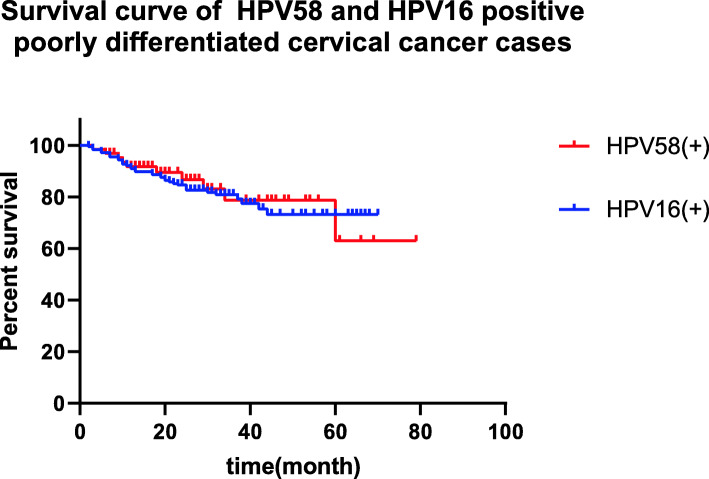
survival curve of poorly differentiated cancer with HPV58 and HPV16 positive cervical cancer cases

Overall survival was not significantly different between the HPV58- and HPV16 positive poorly differentiated cancer groups (63.44±4.50 months vs 57.21±1.93 months, *P*=0.775) (Fig. 1).

### Analysis of stage IA-IIA cervical cancer

#### Clinical features of stage IA-IIA cervical cancer patients

A total of 189 patients with stage II-IIA cervical cancer received surgery. The patients with HPV58-positive cervical cancer with uterine corpus invasion were significantly less than the HPV16 positive patients , accounting for 7.89% and 31.30% respectively (*p*=0.041) (Table 6).

**Table 6:** The clinical feature of 189 cases of stage I- IIA patients with cervical cancer

	HPV58	HPV16	Z	P
Age			0.107	0.744
≤50 years old	18	76		
>50 years old	20	75		
Pathology type			/	1.000
SCC	36	143		
Non-SCC	2	8		
Differentiation			2.980	0.084
Poor	30	97		
Moderately/well	8	54		
Maximum diameter			1.114	0.291
<4 cm	21	69		
≥4 cm	17	82		
Depth of stromal invasion			0.373	0.872
<1/2	14	62		
1/2~2/3	7	28		
>2/3	17	61		
Lymph node metastasis			0.371	0.543
Yes	8	39		
No	30	112		
LVSI			0.106	0.745
Yes	15	64		
No	23	87		
Parametrial invasion			0.988	0.320
Yes	4	9		
No	34	142		
Bladder invasion			/	1.000
Yes	1	4		
No	37	147		
Rectal invasion			/	0.492
Yes	1	2		
No	37	149		
Vaginal invasion			/	0.125
Yes	1	16		
No	37	135		
**Uterine corpus invasion**			/	**0.041**
Yes	3	36		
No	35	115		
Distant metastasis			/	1.000
Yes	0	1		
No	38	150		

#### Comparison of overall survival in patients with and without uterine corpus invasion

**Fig. 2:**
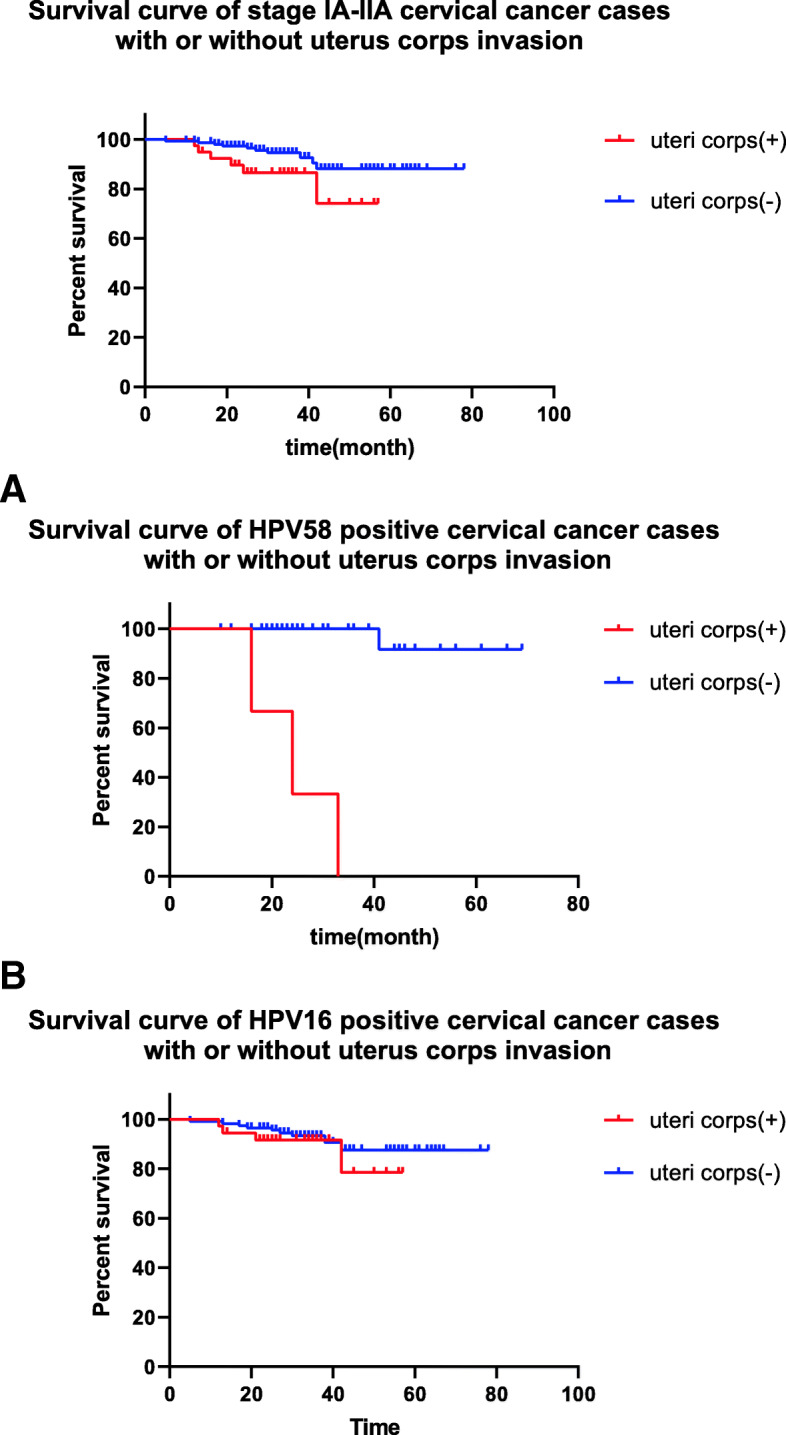
survival curve of stage IA-IIA cervical cancer cases with or without uterus corps invasion. **a**: survival curve of HPV58 positive stage IA-IIA cervical with or without uterus corps invasion. **b**: survival curve of HPV16 positive stage IA-IIA cervical with or without uterus corps invasion

Among patients with stage IA-IIA cervical cancer, despite the HPV genotype, overall survival was significantly lower in patients with uterine corpus invasion than in patients without uterine corpus invasion (49.27±2.72 months vs 72.46±1.78 months, *P*=0.041) (Fig. 2). Further analysis of the data revealed that the overall survival of HPV58-positive cervical cancer patients with uterine corpus invasion was significantly lower than that of without uterine corpus invasion (24.33 months vs 66.67±2.23 months, *P*=0.000) (Fig. 2a). There was no significant difference in overall survival between HPV16 positive cervical cancer patients with and without uterine corpus invasion (51.54±2.59months vs 71.98±2.00months, *P*=0.386) (Fig. 2b).

#### Binary logistic regression analysis for uterine corpus invasion in patients with stage I-IIA cervical cancer

Univariate analysis was performed on the factors affecting uterine corpus invasion to select the significant factors for the binary logistic regression model. HPV58 positivity was an independent risk factor for uterine corpus invasion [HR=3.985, 95% confidence interval: 1.066-14.893, *P*=0.040] (Table 4). Besides , deep stromal invasion and LVSI show a HR of 2.039 [95%CI:1.205-3.452, *P*=0.008] and 0.386 [95%CI:0.164-0.909, *P*=0.029], respectively, which meant HPV58 positivity and deep stromal invasion were the risk factors for uterus corps invasion (HR>1) while LVSI not (HR<1) (Table 7).

**Table 7:** Univariate and multivariate analyses of the risk factors for uterine corpus invasion in stage I-IIA cervical cancer

	Univariate analysis			Multivariate analysis		
	HR	95% CI	P	HR	95% CI	P
HPV58 positivity	3.652	1.060-12.584	**0.040**	3.985	1.066-14.893	**0.040**
Age<50 years old	1.365	0.672-2.774	0.390	/	/	/
Non-SCC	2.426	0.298-19.744	0.408	/	/	/
Moderately/well differentiated	0.484	0.235-0.996	**0.049**	0.531	0.239-1.180	0.120
Maximum diameter>4 cm	1.834	0.885-3.799	0.103	/	/	/
Depth of stromal invasion	2.494	1.569-3.965	**0.000**	2.039	1.205-3.452	**0.008**
Lymph node metastasis (+)	0.280	0.132-0.591	**0.000**	0.636	0.266-1.521	0.309
LVSI (+)	0.236	0.110-0.504	**0.000**	0.386	0.164-0.909	**0.029**
Parametrial invasion (+)	0.383	0.118-1.245	0.111	/	/	/
Bladder invasion (+)	0.162	0.026-1.007	0.051	/	/	/
Rectal invasion (+)	0.514	0.045-5.814	0.590	/	/	/
Vaginal invasion (+)	0.327	0.115-0.923	**0.035**	0.523	0.166-1.645	0.267

### Analysis of stage IIB-IVB cervical cancer

#### Clinical features of patients with stage IIB-IVB cervical cancer

A total of 158 patients with stage IIB-IVB cervical cancer did not receive surgery but received radiotherapy and chemotherapy. The age of the HPV58-positive cervical cancer group was significantly higher than that of the HPV16 positive cervical cancer group (*P*=0.045). Other clinical features are not different from HPV16 positive cervical cancer cases (Table 8).

**Table 8:** Clinical features of 158 patients with advanced stage cervical cancer

	HPV58	HPV16	Z	P
Age			4.034	**0.045**
≤50 years old	9	46		
>50 years old	32	71		
Differentiation			3.657	0.056
Poorly	32	77		
Moderately/well	6	36		
Maximum diameter			0.152	0.696
<4 cm	7	17		
≥4 cm	34	100		
Lymph node metastasis			0.076	0.783
Yes	20	57		
No	21	60		
Bladder invasion			0.682	0.409
Yes	6	24		
No	35	93		
Rectal invasion			/	0.104
Yes	2	18		
No	39	99		
Vaginal invasion			0.286	0.593
Yes	24	74		
No	17	43		
Uterine corpus invasion			0.351	0.553
Yes	19	48		
No	22	69		
Distant metastasis			0.011	0.917
Yes	5	15		
No	36	102		

#### Comparison of overall survival in patients with stage IIB-IVB cervical cancer in different age groups

**Fig. 3:**
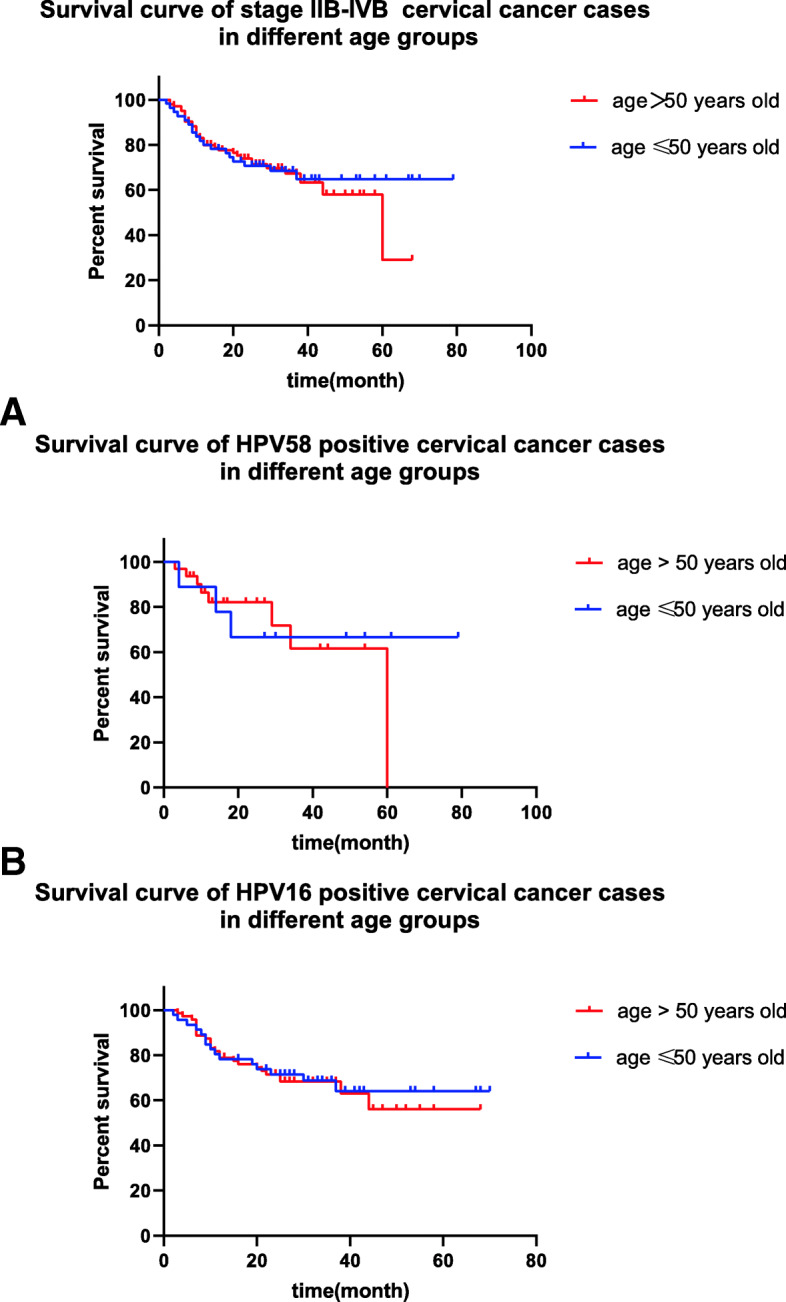
survival curve of stage IIB-IVB cervical cancer cases in different age groups stage. **a**: survival curve of HPV58 positive stage IIB-IVB cervical cancer cases in different age groups stage. **b**. survival curve of HPV16 positive stage IIB-IVB cervical cancer cases in different age groups stage

In spite of different HPV genotypes, the overall survival of patients with stage IIB-IVB cervical cancer in different age groups was not significantly different (56.42±4.38 months vs 45.66±3.20 months, *P*=0.760) (Fig. 3). As to the overall survival of HPV58 positive cases, there was no significant difference between age≤50 and age>50 years old groups (50.48±4.15 months vs 47.09±3.56 months, *P*=0.893) (Fig. 3a). Similarly, the overall survival of HPV16 positive cases was not significantly different between different age groups (56.67±10.59 months vs 44.90±5.19 months, *P*=0.735) (Fig. 3b).

## Discussion

The International Agency for Research on Cancer divided the HPV genotypes into the following groups according to their carcinogenesis: the highly carcinogenic Group 1 (HPV16, 18, 31, 33, 35, 39, 45, 51, 52, 56, 58, and 59); the probably carcinogenic Group 2A (HPV 68); and the possibly carcinogenic Group 2B (HPV26, 30, 34, 53, 66, 67, 69, 70, 73, 82, 85, and 97) [10]. Both HPV58 and HPV16 belong to highly carcinogenic HPV, and they belong to α-9 HPV. HPV16 is more likely to cause cervical cancer [11]. In addition, HPV16 is the most common type of infection worldwide, comparing to HPV31, 33, 35 and 52 [9]. Therefore, this study compared HPV58 positive and HPV16 positive cervical cancer cases to demonstrate the clinical features of HPV58 positive cervical cancer.

Infection with different HPV subtypes may affect cervical cancer prognosis [12, 13], but there still be controversial. Because of the lack of study about HPV58 positive cervical cancer, the overall survival of HPV58 positive cervical cancer remains unclear. Hung-Cheng Lai et al. [12] reported that the prognosis of patients with HPV58-positive cervical cancer was better than that of patients with HPV16 positive cervical cancer. S. Y. Tong et al. [14] demonstrated that HPV genotypes had no effect on the prognosis of cervical cancer. Chyong-Huey Lai et al. [15] revealed that HPV58/33/52 positivity was associated with early stage cervical cancer prognosis. Dong Hang et al. [13] suggested that α-PV9 (including HPV16, 31, 33, 35, 52, 58 and 67) species-related cervical cancer had a better result than cervical cancers associated with other species of HPV. However, clinical stage plays a crucial role in prognosis, and the study did not divide data into different clinical stages. This study shows that there are some distinguishing features between HPV58-positive cervical cancer and HPV16 positive cervical cancer. The percentage of poorly differentiated cancer of HPV58 positive cervical cancer (79.75%) is significantly higher than that of HPV16 (65.57%), and HPV58 positive squamous cells carcinoma is vulnerable to poorly differentiated cancer. But the overall survival of poorly differentiated cancer cases is not significantly different between HPV58 positive and HPV16 positive groups. The result suggests that there are other factors relating to the prognosis of cervical cancer expect the discrepancies of HPV genotypes and pathology grades.

In this study, the proportion of uterine corpus invasion was significantly lower in the HPV58-positive cervical cancer group (7.89%) than in the HPV16 positive group (31.30%). Interestingly, however, multivariate analysis revealed that HPV58 positivity is an independent risk factor for uterine invasion in patients with stage I-IIA cervical cancer (HR 3.985). Meanwhile, deep stromal invasion is an independent risk factor for uterus corps invasion (HR 2.039). Only 3 cases of HPV58 positive stage IA-IIA cervical cancer occur uterus corps invasion, and the 3 cases are with deep stromal invasion (≥2/3 cervix stromal), implying that HPV58 positive cervical cancer in susceptible to uterus corps invasion. But more cases should be included further to confirm that. In addition, all these 3 cases have LVSI, which is consistent with the result of multivariable analysis that LVSI is not the risk factor of uterus corps invasion (HR 0.386). The result is coincident with previous researches. Chen et al [5] pointed out that HPV58 positive cervical cancer was more susceptible to LVSI than HPV16 positive cases, however whether LVSI acted as the risk factor for remain to ambiguous. K. Matsuo and his colleagues [16] reported that older age, larger tumor diameter, poor differentiation, nonsquamous cell carcinoma and lymph node metastasis were risk factors for uterine corpus invasion in early-stage cervical cancer, and uterine body invasion was an independent risk factor for poor prognosis in early cervical cancer. Similarly, Mihai Meirovitz et al. [17] noted that tumor diameter over 2 cm, deep interstitial infiltration (over 5 mm) and lymphovascular space invasion were independent risk factors for extracervical involvement. These results are somewhat similar to those of this study. But it is difficult to speculate whether HPV genotype is a risk factor for uterine corpus invasion because of the lack of studies on the involvement of HPV genotype. According to the results of this study, there are more cases of poorly differentiated cancer in the HPV58 positive cervical cancer group than in the HPV16 positive group. HPV58 is likely associated with poorly differentiated cancer, which makes the tumor more prone to deep stromal invasion. However, further experimental studies are needed. Therefore, for patients with HPV58 positive stage IA-IIA cervical squamous cells carcinoma, who are diagnosed with poorly differentiated cancer and deep stromal invasion, it is necessary to be aware of the possibility of uterine corps invasion.

Despite the HPV genotypes, the overall survival of patients with uterine corpus invasion is significantly shorter than that of patients without uterine corpus invasion (49.27±2.72 months vs 72.46±1.78 months, *P*=0.041). Although HPV58-positive cervical cancer is less likely to have uterine corpus invasion, the prognosis of HPV58-positive cervical cancer with uterine corpus invasion is worse than that of without uterine corpus invasion (24.33 months vs 66.67±2.23 months, *P*=0.000). But it is no significant difference between HPV16 positive cervical cancer cases with or without uterus corps invasion (51.54±2.59months vs 71.98±2.00months, *P*=0.386). According to previous multivariable analysis that shows HPV58 positivity is associated to uterus corps invasion (HR 3.985), it is speculated that HPV genotype is correlated to prognosis of cervical cancer with uterus corps invasion. Uterine corpus invasion is not part of the 2018 FIGO staging system, or the 2019 NCCN guidelines which has been updated recently. The average survival time of patients with uterine corpus invasion and early cervical cancer is 11.2 years, which is significantly lower than that of patients with early cervical cancer but without uterine corpus invasion (14.2 years) [16]. Uterine corpus invasion in early-stage cervical cancer suggests that the tumor is more aggressive [18]. In this study, the 3 cases of HPV58 positive cervical cancer with uterus corps invasion occur deep stromal invasion, which verify the aggression of cervical cancer with uterus corps invasion.

There are a total of 159 cases of stage IIB-IVB cervical cancer. In cases of stage IIB-IVB cervical cancer, the proportion of patients with HPV58-positive cervical cancer over 50 years is significantly higher than that of patients with HPV16 positive cervical cancer. Likely, Hung-Cheng LAI at el [12] got the same result that the age of HPV58 positive cervical cancer patients were older than that of HPV16 positive cases. But the difference of age do not show in the cases of stage IA-IIA. It suggests that HPV58 positive cervical cancer advance more slowly than HPV16 positive and costs more time to advanced stage, which may explain that the patients with HPV58 stage IIB-IVB cervical cancer are older. However, the overall survival of different age groups of stage IIB-IVB cases is not significantly different. Further analysis of HPV58 and HPV16 positive cervical cancer was performed respectively, the similar results were obtained. Although Quinn at el [19]. revealed that the older age is responsible for worse prognosis of cervical cancer patients, it is HR of 1.46 in the age groups of 50-69 years old while that is 2.87 in the age of over 70 years old. The research did not stratify the HPV genotypes, while different HPV genotypes affect the prognosis to some extent, such as HPV18 positive cervical cancer is related to unsatisfied outcome [13], these factors might have effects on the research. Comparing Quinn’s research, our study just includes HPV58 and HPV16 positive cases, which may interpret the inconsistent results.

There are still some limitations in this study. First, this is a single-center retrospective analysis with a small sample size. Second, the disease-free survival data are incomplete and were not included in the results of this study. The assessment of recurrence could not be performed.

## Conclusion

In conclusion, regardless of clinical stage, HPV58-positive cervical cancer patients are at higher risk of poorly differentiated cancer, but it is no related to the prognosis. Although the patients with early stage HPV58-positive cervical cancer are not easy to occur uterus corps invasion, the prognosis is worse than that without uterus corps invasion. As to the HPV58 positive early stage cervical squamous cells carcinoma patients who are diagnosed with poorly differentiated cancer and deep stromal invasion should be vigilant to uterus corps invasion. And advanced-stage HPV58-positive cervical cancer is more common in patients over 50 years old than in patients 50 years or younger.

## Data Availability

The datasets used and/or analyzed during the current study are available from the corresponding author on reasonable request.
